# Mass drug administration for lymphatic filariasis elimination in a coastal state of India: a study on barriers to coverage and compliance

**DOI:** 10.1186/2049-9957-3-31

**Published:** 2014-09-01

**Authors:** Mohammad A Hussain, Ashok K Sitha, Subhashisa Swain, Shridhar Kadam, Sanghamitra Pati

**Affiliations:** 1 School of Population Health, The University of Queensland, Herston, Brisbane, QLD 4006, Australia; 2 Government of Odisha, Odisha, India; 3 Public Health Foundation of India, Indian Institute of Public Health, Bhubaneswar, Odisha, India

**Keywords:** Lymphatic filariasis, Mass drug administration, Compliance, Consumption, Elimination

## Abstract

**Background:**

Lymphatic filariasis is targeted for elimination in India through mass drug administration (MDA) with diethylcarbamazine (DEC) combined with albendazole (ABZ). For the strategy to be effective, >65% of those living in endemic areas must be covered by and compliant to MDA. Post the MDA 2011 campaign in the endemic district of Odisha, we conducted a survey to assess: (i) the filariasis knowledge in the community, (ii) the coverage and compliance of MDA from the community perspective, and (iii) factors affecting compliance, as well as the operational issues involved in carrying out MDA activities from the drug distributor’s perspective.

**Methods:**

A sample of 691 participants – both male and female, aged two years or above – were selected through multistage stratified sampling and interviewed using a semi-structured questionnaire. Additionally, drug distributors and the medical officers in charge of the MDA were also interviewed to understand some of the operational issues encountered during MDA.

**Results:**

Ninety-nine percent of the study participants received DEC and ABZ tablets during MDA, of which only just above a quarter actually consumed the drugs. The cause of non-compliance was mostly due to fear of side effects, lack of awareness of the benefits of MDA, and non-attendance of health staff in the villages. Lack of adequate training of drug distributors and poor health communication activities before the MDA campaign commenced and the absence of follow-up by health workers following MDA were a few of the operational difficulties encountered during the MDA campaign.

**Conclusion:**

Currently MDA is restricted to the distribution of drugs only and the key issues of implementation in compliance, health education, managing side effects, and logistics are not given enough attention. It is therefore essential to address the issues linked to low compliance to make the program more efficient and achieve the goal of filariasis elimination.

## Multilingual abstracts

Please see Additional file 1 for translations of the abstract into the six official working languages of the United Nations.

## Background

Lymphatic filariasis (LF) or elephantiasis is one of the six diseases that can potentially be eradicated [1]. It is the fourth most common cause of disability worldwide [2]. The infection is endemic in more than 80 countries, with more than 1.3 billion people at risk and 120 million already infected globally [1]. Two-thirds of the endemic population resides in South-East Asia and one-third lives in India [3]. Considering the human suffering, social stigma and costs associated with LF morbidity, and in response to the specific resolution by the World Health Assembly, the Global Program to Eliminate Lymphatic Filariasis (GPELF) was launched by the World Health Organization (WHO) in 2000 with the goal of eliminating LF as a public health problem by the year 2020 [4].

In 2002, India set an ambitious national health goal to eliminate LF by 2015 [5]. In order to achieve this goal, a “two-pillars” strategy of interrupting transmission through mass drug administration (MDA) with diethylcarbamazine (DEC) and providing care for those with the disease was adopted [6].

India’s filarial control program has scaled up MDA over the past several years and recently added albendazole (ABZ) to the treatment of the 590 million Indians living at risk of infection [7]. The principle behind MDA is that a single dose of DEC administered annually continued for four to six years will interrupt the transmission of filariasis [8]. Micro-simulation models showing the effect of MDA on LF elimination demonstrate that the number of MDA rounds necessary to achieve elimination depends, to a large extent, on coverage, drug efficacy, and the endemicity level [9] Countries such as India have additional challenges due to the many diverse geographical areas with high endemicities of filariasis, the populations of which will require more rounds of MDA. Despite this, the program has been successful in distributing a sufficient number of tablets to the high-risk population. However, ensuring adherence and compliance to the regimen is still a problem in many parts of country [10,11].

In India, the coverage levels varied from 55% to 90% [12]. When a proportion of the population fails to comply with MDA, a potential reservoir for the parasite is left untreated, opening the door to recrudescence of the microfilaraemia (mf) and thus reducing the probability of the program’s success [13]. It is estimated that in order to interrupt transmission, MDA compliance must exceed 65–75%, with five to six rounds of treatment [9], however, compliance is relatively low in the majority of the endemic areas [10,14,15]. Odisha has reported an mf rate of 0.43 in 2011 compared to 2.57 in 2004 [16]. However, coastal districts are more endemic for the disease, particularly the district Puri. Considering the fact that many of Odisha’s non-coastal districts were non-endemic for filarial, this reported mf rate could be misleading.

In support of this, various research has reported low compliance rates in endemic districts [17-19]. Studies have shown that various factors responsible for compliance varied according to the geographical area and health system function [20,21]. Many studies have been done to find out the reasons from the community perspective, but very few made attempts to understand the operational issues from the distributor’s perspective. The roles of the drug distributors and other health workers cannot be ignored in order to achieve success in MDA coverage and compliance. Keeping this in mind, this cross-sectional survey was carried out in the Puri district of Odisha state in India. The recent round of MDA activity prior to the study was carried out in March 2011. Intensive behaviour change communication (BCC) aactivities were carried out to make the community aware of the facts pertaining to LF and cooperate in the consumption of the drug. We conducted the study from April 1 to June 30, 2011, with the objectives to assess the knowledge of filariasis, the coverage and compliance of MDA during this period, and to explore the factors affecting compliance and the operational issues involved in carrying out MDA activities from the perspective of the medical officers and drug distributors.

## Methods

### Study area

The district Puri situated on the coast of the Bay of Bengal is one of the highly endemic districts for LF and was considered to have low coverage of MDA since 2002. Presently, around 1.5 million people are at risk of filarial infection in the district. The district is divided administratively into 11 blocks, out of which four are predominantly urban and seven are rural.

### Study design, setting, and participants

To achieve the framed objectives, three different categories of study participants were selected. For the assessment of coverage and compliance, a representative sample of individuals aged two years or above was selected. Any individual severely ill or pregnant was excluded. Furthermore, to have an idea about the operational issues or problems during the drug distribution, the medical officer in charge of the community health center (situated in the selected blocks and responsible for all program activities) who was responsible for the MDA and one health worker from each sampled area were also interviewed.

### Sample size and sampling technique

The sample size was calculated based on the previous reported compliance of MDA in the study district [12]. Taking into consideration the MDA compliance in the study district to be 72%, with an absolute precision of 5% and at a 5% level of significance with a design effect of two, the sample size was calculated to be 620. Assuming a non-response rate of 10%, the final sample size was rounded up to 691 calculated using OpenEpi, version 2.3.1 [22]. An equal proportion of participants was selected from each block and included in the study through multistage sampling. Initially, a list of all the villages in each block, as well as the wards in each urban administrative unit, was prepared and one village/ward was selected randomly from each block for the selection of the study respondents. In total, seven villages and four urban wards were selected representing each administrative block.

Secondly, the first household was selected as per the extended program for immunization EPI recommendation [23], and thereafter the fifth nearest household was selected to give a widespread coverage and adequate representation [24]. All the eligible members of the household were interviewed about coverage and compliance. If someone was absent during the day of the interview, this person’s information was not collected. The children below 10 years of age were not assessed for knowledge of LF. For those children who were not sure about the receipt and consumption of drugs, the information was ascertained from their mothers or any available adult family member who was aware of their consumption status. If one village was not covered in one day then a second visit was made to complete the interviews. In total, 11 medical officers and 11 drug distributors/health workers were included in the study.

### Data collection and study variables

Data was collected with the help of an interviewer-administered questionnaire containing both open- and closed-ended questions. All interviews were conducted by one of the authors, AKS, who is a trained medical graduate and well versed in the local health system, as well as the local communities. Three different sets of questionnaires were used, one for the individual respondents (eligible representative sample), one for the drug distributors, and one for the medical officers. Items and possible options for the questions were decided upon based on the personal experiences of the researchers, from available published literature, and from a pilot qualitative study done prior to this study.

The assessment of the coverage and compliance was for the round of MDA conducted in March 2011. Since the data collection for the study was done during April and May 2011, which was one month after the MDA round, the chances of experiencing recall bias was assumed to be less. For all practical purposes, coverage and compliance were defined as follows:

Coverage was defined as the percentage of eligible individuals who received the drugs DEC and ABZ distributed during the MDA in March 2011. Compliance was defined as the percentage of individuals who self-reported that they consumed the drugs, among those who received the drugs.

The socio-demographic variables studied were age, sex, education, occupation, and place of residence. In relation to the compliance, data was obtained on the number of tablets taken, whether the drug was taken under direct observation of the drug administrator, any side effects experienced, and the reasons for non-compliance.

The respondents’ basic knowledge of filariasis, particularly its causative agent, signs and symptoms, treatment options, individual perception about susceptibility to filarial infection and awareness about MDA, were also explored. Similarly, the questionnaire for the health workers/drug distributors was designed to elicit how they procured and distributed drug, what they advised patients, and the problems they faced during drug distribution. The questionnaire for the medical officers attempted to explore the operational difficulties during the MDA program.

### Data entry and analysis

The data from the household survey form was entered into MS Excel spreadsheet and analyzed using SPSS version 15.0.

The coverage and compliance were calculated in percentages and their population estimates were expressed in terms of Fleiss quadratic 95% confidence interval (CI) using Epi Info 6. Other details of compliance and non-compliance were also expressed as percentages. The chi-square test was done to assess the significance of the socio-demographic and other variables related to compliance. The qualitative data (obtained using an open-ended questionnaire) were analyzed using the “*thematic framework”* approach. While synthesizing both the qualitative and quantitative data, various themes that influenced coverage and compliance of MDA were identified and categorized. These included health system/policy, and community- and drug-related issues. Ethical permission for the study was obtained from the Institutional Ethical Committee of the Indian Institute of Public Health, Bhubaneswar, prior to the study. Written and verbal consent was obtained from all study participants before they were included in the study.

## Results

### Background characteristics

Out of 691 study participants, males and females were nearly equally distributed. About 59% (95% CI: 54.6%–62.0%) of the participants were in the age group of 16 to 45 years. The literacy rate among males was higher than among the females. Individuals from rural areas comprised 68.7% of the sample (see Table 1).

**Table 1:** Socio-demographic characteristics of the study participants

***Variables***	***Categories***	***Male (N = 334) % (95% CI)***	***Female (N = 357) % (95% CI)***	***Total (N = 691) % (95% CI*)**
*Age group*	3–10	5.38(3.32–8.22)	8.75(6.13–12.05)	7.09(5.35–9.19)
	11–15	6.28(4.03–9.29)	15.53(12.0–19.6)	11(8.8–13.5)
	16–45	59(53.6–64.1)	58.2(53–63.2)	58.3(54.6–62.0)
	>45	29.3(24.6–34.4)	18.3(14.6–22.6)	23.6(20.5–26.9)
*Education*	Illiterate	3.9(2.18–6.4)	6.7(4.5–9.7)	5.3(3.8–7.22)
	3–7 years	7.2(4.7–10.3)	27.4(22.9–32.2)	17.5(14.8–20.5)
	7–10 years	17.3(15.6–21.7)	22.3(18.2–26.8)	19.8(17.0–23.0)
	>10 Years	71.5(66.5–76.2)	44.35(39.2–49.5)	57.3(53.6–61.0)
*Area*	Urban	57.9(51.2–64.3)	41.12(35.6–48.8)	31.3(27.9–34.7)
	Rural	50.5(46.0–55.0)	49.4(45.0–54.0)	68.7(65.2–72.1)

### Knowledge about filariasis

The study participants demonstrated a good knowledge of filariasis, as well as of MDA. Nearly 84% (95% CI: 80.7%–86.3%) considered themselves to be at risk of getting disease and 88% (95% CI: 85.2%–90.1%) cited mosquito bites as the mode of transmission (see Table 2). A similar response was obtained when participants identified the symptoms and treatment for filariasis. We could not find any association between knowledge and drug consumption during the MDA program.

**Table 2:** Knowledge of filariasis in the community

***Variables***	***Responses***	***Male (N = 334) % (95% CI)***	***Female (N = 357) % (95% CI)***	***Total (N = 691) % (95% CI)***
What is filariasis?	Elephantiasis	61.6(65.4–74.8)	70.3(65.4–74.8)	67.8(64.3–71.3)
	Lymphedema and elephantiasis	32.5(27.7–37.5)	29.1(24.6–34.0)	31.7(28.3–35.2)
	Do not know	0.3(0.01–1.4)	0.56(0.1–1.70)	0.43(0.11–1.17)
Mode of transmission for filariasis?	Mosquito bite	90.4(86.9–93.2)	85.4(81.5–88.8)	87.8(85.2–90.1)
	Hereditary/contagious	8.7(6.0–12.0)	13.1(9.9–16.5)	11(8.8–13.5)
	Do not know	0.9(0.22–2.42)	1.4(0.5–3.07)	1.15(0.54–2.2)
Most common symptoms	Swelling of limbs	60.4(55.2–65.7)	34.0(29.1–39.0)	46.7(43.0–50.5)
	Fever and swelling	38.6(33.5–44.0)	65.2(60.2–70.0)	52.4(48.6–56.1)
	Do not know	0.9(0.22–2.42)	1.12(0.35–2.68)	1.0(0.44–2.0)
Treatment	Medicine	90.1(86.5–93.0)	85.0(80.5–88.0)	87.3(84.6–89.6)
	Not curable	8.9(6.25–12.42)	13.4(10.2–17.3)	11.3(9.1–13.8)
	Do not know	0.9(0.22–2.42)	1.96(0.9–3.8)	1.4(0.7–2.56)
Knowledge regarding the distribution of filarial tablets for prevention	Yes	95.5(92.9–97.4)	90.5(87.0–93.2)	92.9(90.8–94.6)
	No	4.5(2.63–7.13)	9.5(6.8–12.9)	7.1(5.35–9.20)
Do you consider yourself to be at risk?	Yes	86.5(82.5–89.9)	80.9(76.6–84.7)	83.6(80.7–86.3)
	No	12.3(9.0–16.13)	17.08(13.4–21.2)	14.7(12.2–17.5)
	Do not know	1.2(0.4–2.9)	1.96(0.9–3.8)	1.6(0.83–2.75)

### Coverage and consumption

**Figure 1:**
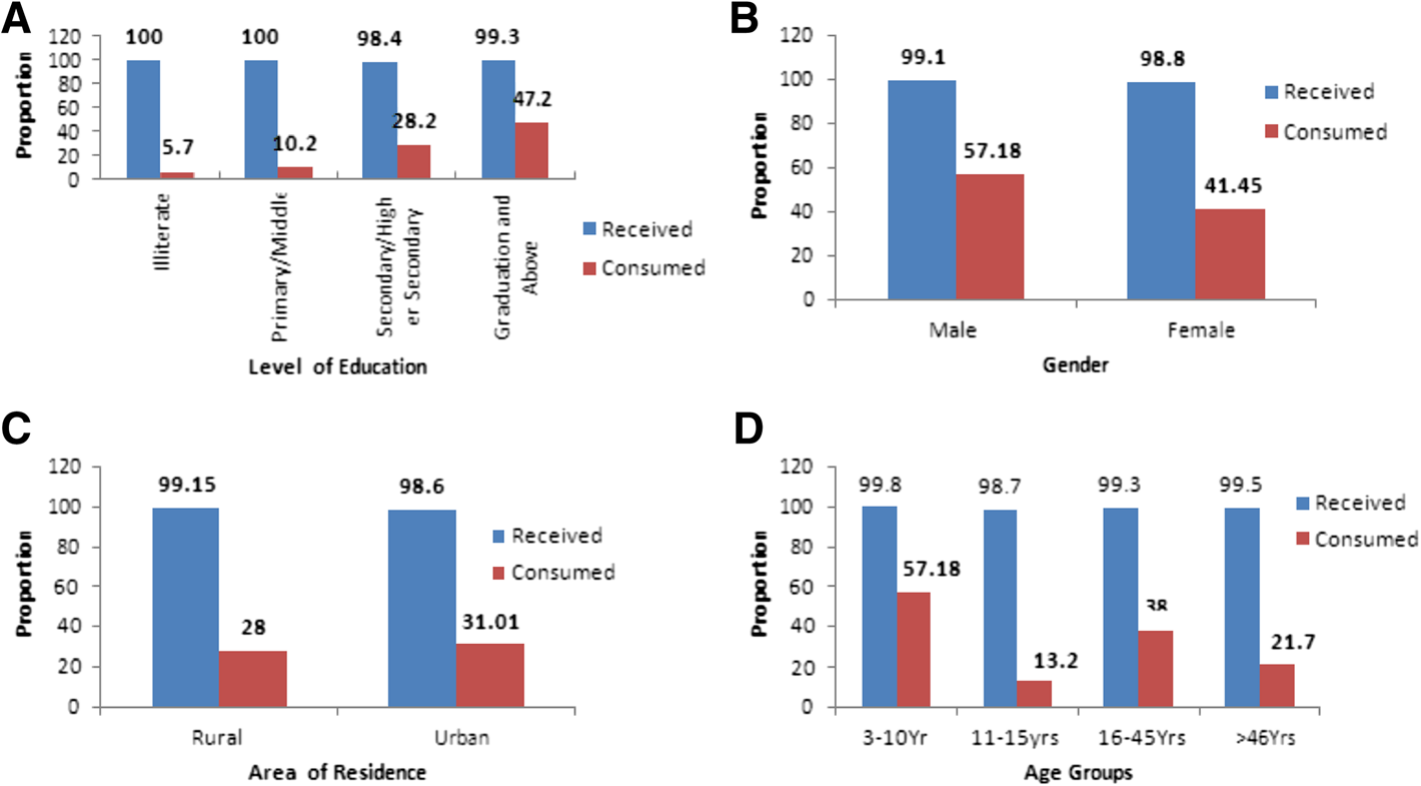
**Coverage and compliance of DEC and albendazole according to various demographic characteristics.** Significant difference (Chi square test, p<0.05) in consumption pattern was observed across different groups. Coverage in compliance according to **(A)**-Level of Education; **(B)**-Gender; **(C)**-Area of residence; **(D)**-Different age groups.

**Figure 2:**
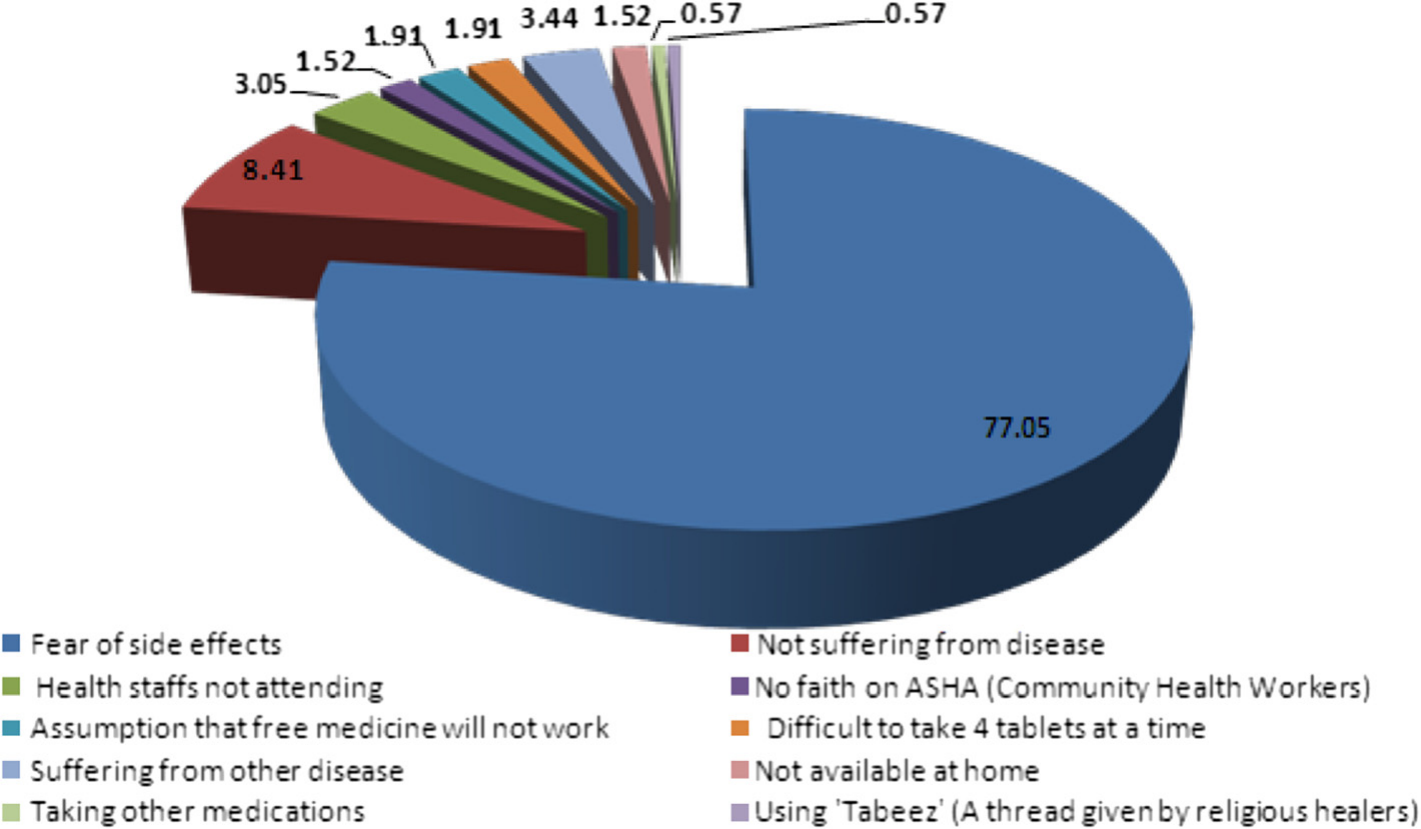
Reasons of non-consumption of DEC by the respondents.

**Figure 3:**
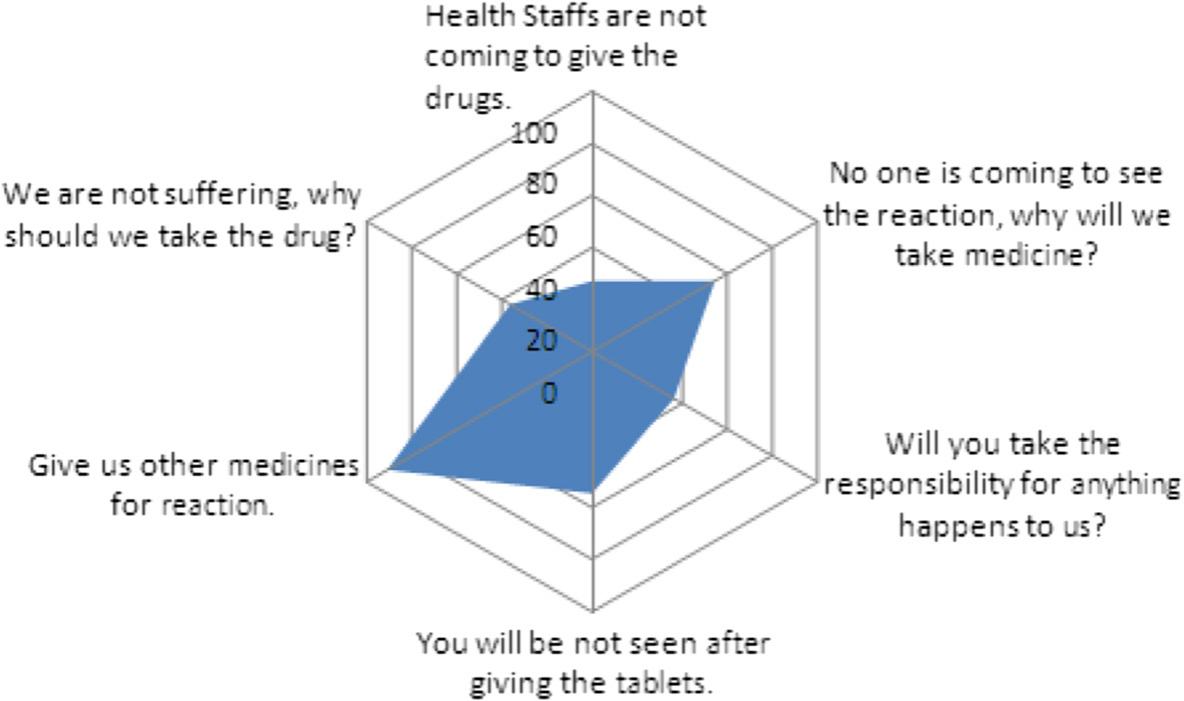
Major reasons for non-consumption of DEC as qouted by individuals during in depth discussions.

Nearly 99% of the studied individuals in both rural and urban areas received DEC and ABZ during the MDA campaign. However, less than a third (28% in the rural areas and 31% in the urban areas) had consumed the distributed drugs. There was a significant heterogeneity (p < 0.001) in the consumption pattern among males and females with a higher proportion of males consuming the drugs than females, and also across the educational status and age group (see Figure 1). No difference was observed in the consumption pattern among the residents in rural and urban areas (p = 0.432). The various reasons expressed by the study participants for non-consumption are depicted in Figure 2. Out of all of them, the fear of side effects (77%) was the major cause for non-consumption. The reasons for non-consumption were probed further and the major responses are depicted in Figure 3.

### Problems faced by the health staff

The discussions with the health workers/drug distributors highlighted some operational issues, including non-availability of medicine for side effects and difficulties in distributing the medicine to everyone in one day, which pose obstacles in carrying out the MDA activities. Non-operationalization of behavior change communication activities before drug distribution, no follow-up of health workers/drug distributors to ascertain drug intake, inadequate training of drug distributors, delay in receiving funding for MDA activities, and supervisors not performing their responsibilities properly were a few of the operational difficulties cited by the concerned medical officers. Both the medical officer in charge of the MDA program and the health workers perceived that the unwillingness of people to take the medicine was a major hindrance in the effective implementation of this program.

People consider elephantiasis to be synonymous with LF and mentioned that they were not observing many lymphoedema cases in their locality. Hence, some of the participants felt that they were not at risk of getting the disease. One of the respondents said: “*There isn’t a filarial patient in our house, so it is not going to spread in our home. Two filaria patients in our village are from the same family and as they are residing at the extreme end of the village, the infection will not attack us.”*

The most common reason cited for not adhering to DEC was the fear of side effects. One of the ladies stated: “*I would have died if I was not admitted to the headquarter hospital during the night after consuming the tablets. There was no one to look after me. After the Anganwadi worker gave me the medicine, I could not find him again. There was no one in our nearby hospital when I arrived there.”*

Similarly another lady stated: *“I was afraid of what would happen during the night after consuming the tablet, so I did not consume the tablet and I did not give it to other family members. A few years back, somewhere someone died after consuming the tablets.”*

### Barriers at the distribution level

Medical officers in charge expressed difficulties in the operationalization of the program. One medical officer said: *“The message for the purpose of MDA in the village is not reach the people properly. This is the main cause of non-consumption. The training should be given way before the program commences. We did the training quickly, so we could not do the program justice.”*

## Discussion and Conclusion

The present study indicated that even though the coverage of the MDA in Odisha was 99%, compliance was relatively low. Mathematical models predict that an annual mass treatment of four to six years is required for the elimination of LF, assuming that the reproductive life span of *Wuchereria bancrofti* is approximately five years with a coverage rate of 65% [25,26].

In our study, there is difference in compliance between males and females, as well as between the different age groups. This difference could be due to a higher literacy level among males as compared to the females. A detailed probe into the matter might help us determine the other cultural and social factors related to gender which could have played a role in the compliance rate. One reason for the increased compliance in males is that the drugs might have been distributed in schools and students were instructed by their teachers to consume the tablets. No doubt, awareness and education play a crucial role in the elimination of LF [17]. The present study has identified certain aspects in which people need to be sensitized and educated. Many respondents thought that the disease manifested only in the form of elephantitis and few knew that LF is vertically transmitted.

The incidence of side effect estimates ranged from 25.4% to 82.1% in India [9,19], however, the majority of the side effects were mild and infrequent [9,14]. Side effects decline with the subsequent rounds of MDA [24]. In our study, the fear of side effects was the major issue despite good coverage. A similar result was seen in previous studies conducted in India, but the current program has failed to solve this issue and gain faith among individuals. An educational message describing the most frequent, mild side effects along with simple suggestions on how to manage them could alleviate fear and thus increase MDA compliance. The educational message should also indicate that many of the side effects are related to the killing of LF by DEC and that the probability of side effects decreases with the number of microfilaria also decreasing. A strong post distribution follow-up mechanism might be helpful to achieve better compliance.

The second barrier to compliance identified in the MDA program was that many people still did not recognize the benefits of the medication. This is neither a new nor a surprising finding [10,14]. Although the side effects of DEC have been highly publicized, the benefits of the drug have not been described as well. As approximately two-thirds of those infected remain asymptomatic, these individuals in particular may not realize that they could personally benefit from DEC. The message that all people living in endemic areas are at risk of infection and that one could be infected even if asymptomatic should be emphasized in upcoming pre-MDA educational campaigns. Challenges from the distributor’s perspective also need to be addressed. Operational problems such as lack of timely funding, inadequate training, and no IEC activities need to be focused upon.

The third problem cited was the lack of availability of health workers after drug distribution and the absence of follow-up. The predominant reason for not receiving tablets was that the distributor did not revisit the houses. This can also be avoided. Even further, follow-up of the individuals to observe side effects and increase awareness can be done with little extra effort. Lack of time to cover and revisit the houses, lack of medicine for side effects (even though they are minimal), and difficulty in receiving the drugs from health centers were found to be hampering the process. There is a need for an integrated approach connecting health workers, policymakers, and the community to address the problem more comprehensively. The inclusion of surveillance of adverse reactions after MDA can be a proxy indicator for the consumption rate. Issues related to the health system in procurement policy have influenced the coverage and compliance of MDA in Odisha. The problem at the planning level can be solved with a proper mobilization of resources and advocacy.

We found a similar result reported by Showkat et al. [20] and Kumar et al. [27] regarding MDA compliance. That study was done in Kerala, the state with highest literacy rate (99%). Even though the literacy rate is higher in Kerala, the acceptance of MDA was still low [20]. A systematic review published in 2012 cited similar problems affecting the compliance rate globally, which demands the health system to get involved and further research to be conducted in order to address the problem [21]. This study was cross-sectional in nature, which inherits the possible biases of responses. We had not probed in detail about cultural and social barriers. Responses obtained from children have to be considered carefully.

### Policy implications

Strengthening the IEC in rural as well as in urban areas focusing on the prevention of filariasis can be done once a year. Sensitizating the populations about the risk and benefits of DEC is essential. Supportive supervision and monitoring of activities need to be strengthened and focused on in the program. Mobilization of the community, proper training in line with the aim of the program, and how and what type of information is given to communities has to be addressed. On-the-spot administration and surveillance for adverse reactions can enhance the compliance rates. Despite a significant reduction in the mf rate from 2.5 to 0.4, the complete elimination of filariasis in Odisha depends on the sustained pursuance of MDA coverage coupled with compliance. However, this data is for the entire state and district specific interventions for the endemic areas are urgently needed. Vigorous surveillance, advocacy, and community-based education are also essential tools in making this goal a near reality.

### Ethical approval

Ethical permission was obtained from the Institutional Ethics Committee, Indian Institute of Public Health, Bhubaneswar.

## Competing interests

The authors declare that they have no competing interests.

## Authors’ contributions

MAH and AKS conceived the study. MAH, AKS, SP, and SK designed the study protocol. AKS collected the data. MHA and SS provided assistance for all experiments in the analysis and in the drafting of the paper. All authors read and approved the content of the manuscript.

## Supplementary Material

Additional file 1 Multilingual abstracts in the six official working languages of the United Nations. Click here for file
