# Supplementary material for: Mass drug administration for lymphatic filariasis elimination in a coastal state of India: a study on barriers to coverage and compliance
Source: Infect Dis Poverty. 2014 Sep 1;3:31. doi: 10.1186/2049-9957-3-31 (PMC4166397; doi:10.1186/2049-9957-3-31)
Supplement: Additional file 1 — Multilingual abstracts in the six official working languages of the United Nations. [file 2049-9957-3-31-S1.pdf]

Translation of the abstract into the six official working languages of the United Nations

العلاج الجماعي بالدواء للقضاء على الفلاريا الليمفاوية بولاية ساحلية في الهند: دراسة عن العوائق التي تحول دون التغطية والامتثال

محمد أ. حسين، أشوك ك. سيتها، سباششيسا سوين، شريدهار كدام، سانجامترا باتي

الملخص

الخلفية: يستهدف للقضاء على الفلاريا الليمفاوية في الهند من خلال العلاج الجماعي بالدواء (MDA) باستخدام ثنائي إيثيل كاربامازين (DEC) جنباً إلى جنب مع البندازول (ABZ). ولتكون الإستراتيجية فعالة، فإن أكثر من 65% من أولئك الذين يعيشون في المناطق الموبوءة يجب أن تغطى وتمتثل للعلاج الجماعي بالدواء. بعد حملة 2011 للعلاج الجماعي بالدواء بمقاطعة أوديشا الموبوءة، أجرينا مسحاً لتقييم: (i) مدي الدراية بالفلاريا في المجتمع، (ii) التغطية والامتثال للعلاج الجماعي بالدواء من وجهة نظر المجتمع، و (iii) العوامل التي تؤثر على الامتثال، بالإضافة إلى الأمور التشغيلية المستخدمة في تنفيذ أنشطة العلاج الجماعي بالدواء من وجهة نظر موزع الدواء.

طرق العمل: تم اختيار عينة من 691 مشارك – ذكوراً وإناثاً، تتراوح أعمارهم بين عامين أو أكثر – من خلال أخذ عينة طبقية متعددة المراحل ومقابلات باستخدام استبيان شبه كامل. بالإضافة إلى ذلك، أجريت أيضاً مقابلات مع موزعي الدواء والمسئول الطبي المعنيين بالعلاج الجماعي بالدواء ليفهموا بعض الأمور التشغيلية التي واجهتهم أثناء العلاج الجماعي بالدواء.

النتائج: تلقى تسعة وتسعين بالمائة من المشاركين في الدراسة أقراص ثنائي إيثيل كاربامازين والبندازول أثناء العلاج الجماعي بالدواء، تناول فقط أكثر من ربعهم الدواء فعلياً. وكان سبب عدم الامتثال على الأغلب بسبب الخوف من الآثار الجانبية، وقلة الوعي بفوائد العلاج الجماعي بالدواء، وعدم حضور الموظفين الصحيين في القرى. نقص التدريب الملائم لموزعي الدواء وقلة أنشطة الاتصال الصحية قبل بدء حملة العلاج الجماعي بالدواء وغياب المتابعة من قبل العاملين الصحيين التابعين للعلاج الجماعي بالدواء كانت قليل من الصعوبات التشغيلية التي تم مواجهتها أثناء حملة العلاج الجماعي بالدواء.

الاستنتاج: يقتصر العلاج الجماعي بالدواء حالياً على توزيع الدواء فقط والقضايا الرئيسية الخاصة بتنفيذ الامتثال، والتثقيف الصحي، والتعامل مع الآثار الجانبية والخدمات اللوجستية لا تعطى اهتماماً كافياً. ولذا فمن الضروري معالجة القضايا المرتبطة بانخفاض الامتثال لجعل البرنامج أكثر فعالية وتحقيق هدف القضاء على الفلاريا.

Translated from English version into Arabic by Saher Salama, through

TRANSLATORS
WITHOUT BORDERS

印度沿海地区采用群体治疗消除淋巴丝虫病：覆盖面与依从性研究

Mohammad A. Hussain, Ashok K. Sitha, Subhashisa Swain, Shridhar Kadam, Sanghamitra Pati

摘要：

引言：印度旨在通过乙胺嗪联合阿苯达唑群体治疗（MDA）来消除淋巴丝虫病。为保证其有效性，MDA必须覆盖流行区65%以上的居民，并保证依从性。在流行区奥里萨邦进行的MDA 2011年行动中，开展了一项调查，用于评估以下几方面内容：(1) 人群的丝虫病知识；(2) 从社区角度评价MDA的覆盖情况和依从性；(3) 影响依从性的因素，同时还有包括药物发放员开展MDA相关活动在内的操作问题等。

方法：通过多阶段分层采样的方法选择了691位调查对象（男女均有，不小于2岁），并采用半开放式问卷进行访谈。此外，访谈负责MDA的药物发放员和卫生官员，了解在整个过程中遇到的操作问题。

结果：99%的调查对象拿到了乙胺嗪和阿苯达唑片，但实际上仅有1/4的人服药。依从性差的原因主要是担心药物的不良反应，缺乏对MDA益处的了解，以及在乡村缺少医疗工作者。MDA行动开始前对药物发放员和健康宣传活动的培训不够，并且在MDA行动后没有医疗工作者进行随访，这是MDA行动所遇到的一些困难。

结论：目前群体治疗仅局限于药物发放，而依从性、健康教育、药物不良反应的处理和物流管理等关键问题尚未引起足够的重视。因此，必须解决这些与低依从性相关的问题，从而保障项目更加有效的实施，最终实现消除丝虫病。

Translated from English version into Chinese by Yin Jian-hai, through

CDC CHINESE CENTER FOR DISEASE CONTROL AND PREVENTION

NATIONAL INSTITUTE FOR PARASITIC DISEASES

Administration massive de médicaments destinée à éliminer la filariose lymphatique dans un état côtier de l'Inde : une étude sur les obstacles à la couverture et à l'observance

Mohammad A. Hussain, Ashok K. Sitha, Subhashisa Swain, Shridhar Kadam, Sanghamitra Pati

Résumé

Contexte : En Inde, la filariose lymphatique est la cible d'une administration massive de médicaments (AMM), à base de diéthylcarbamazine (DEC) combinée à l'albendazole (ABZ), destinée à l'éliminer. Pour que la stratégie soit efficace, > 65 % des personnes vivant dans les zones endémiques doivent être couvertes par l'AMM et se conformer au traitement. Après la campagne d'AMM en 2011 dans le district endémique d'Odisha, nous avons mené une étude visant à évaluer : (i) la connaissance de la filariose dans la communauté, (ii) la couverture et l'observance de l'AMM du point de vue de la communauté, et (iii) les facteurs affectant l'observance, ainsi que les problèmes opérationnels liés à la réalisation des activités d'AMM du point de vue des distributeurs de médicaments.

Méthodes : Un échantillon de 691 participants - hommes et femmes, âgés de deux ans et plus - ont été sélectionnés par échantillonnage stratifié à plusieurs degrés et interrogés à l'aide d'un questionnaire semi-structuré. En outre, les distributeurs de médicaments et le médecin responsable de l'AMM ont également été interrogés, dans le but de comprendre certains des problèmes opérationnels rencontrés au cours de l'AMM.

Résultats : Quatre-vingt-dix-neuf pour cent des participants à l'étude ont reçu les comprimés de DEC et ABZ au cours de l'AMM, à peine plus d'un quart d'entre eux ayant effectivement pris les médicaments. Les causes de non-observance étaient principalement liées à la peur d'effets secondaires, au manque de prise de conscience des avantages de l'AMM, et à l'absence de personnel de santé dans les villages. Le manque de formation adéquate des distributeurs de médicaments, les manquements des activités de communication de santé précédant le début de la campagne d'AMM et l'absence de suivi par les agents de santé après l'AMM, étaient quelques-unes des difficultés opérationnelles rencontrées au cours de la campagne d'AMM.

Conclusion : Actuellement, l'AMM se limite à la seule distribution de médicaments, et les questions clés de la mise en œuvre de l'observance, de l'éducation sanitaire, de la

gestion des effets secondaires, et de la logistique, ne reçoivent pas assez d'attention. Il est donc essentiel d'aborder les questions liées à la faible observance pour rendre le programme plus efficace et atteindre l'objectif, l'élimination de la filariose.

Translated from English version into French by Jacek Sierakowski, through

TRANSLATORS WITHOUT BORDERS

Массовые терапии для предотвращения лимфатического филяриаза в прибрежном штате Индии: изучение препятствий для массового покрытия и причин несоблюдения терапевтических рекомендаций.

Мохаммед А Хуссейн, Ашок К Ситха, Субхашиса Свейн, Шридхар Кадам, Сангамитра Пати.

Синопсис

Общая информация: Лимфатический филяриаз в Индии предполагается искоренить путем массовой лекарственной терапии диетилкарбамазином (DEC) совместно с альбендазолом (ABZ). Для того, чтобы терапия была успешной, >65% населения в эндемичных районах должны принять участие в терапии и соблюдать терапевтические рекомендации. После окончания кампании по массовой лекарственной терапии в 2011 году в эндемическом районе Одиша, мы провели опрос для оценки: (i) осведомленности местного населения о филяриозе, (ii) области покрытия и соблюдения терапевтических рекомендаций с точки зрения местного населения, а также (iii) факторов, препятствующих соблюдению терапевтических рекомендаций, и организационных проблем массовой терапии с точки зрения распространителей препарата.

Методология: Выборка из 691 участников – женщин и мужчин, старще двух лет – была сделана с помощью многоступенчатого стратифицированного отбора. Интервью были проведены по слабоструктурированному опроснику. Также были проведены интервью с распространителями лекарственных препаратов и операционным менеджером по массовой терапии для того, чтобы более наглядно представить себе трудности, связанные с организацией массовой терапии.

Результаты: Девяносто шесть процентов респондентов в исследовании получили препараты DEC и ABZ во время массовой терапии, но только немногим более четверти опрошенных приняли препараты. Причины несоблюдения терапевтических рекомендации явились боязнь побочных эффектов, неосведомленность о значении массовой терапии и непосещение медицинских центров в населенных пунктах. Основными трудностями, препятствующими успешному проведению массовой лекарственной терапии явились недостаточная предварительная подготовка распространителей препаратов и отсутствие медицинской поддержки по завершению кампании.

Заключение: В настоящее время массовая терапия занимается только

распространением лекарственных препаратов, но такие ключевые факторы, как соблюдение терапевтических рекомендаций, распространение информации о здравоохранении, возможные побочные эффекты и логистика, почти не принимаются во внимание. Необходимо обратить пристальное внимание на проблемы несоблюдения или неадекватного соблюдения терапевтических рекомендаций, чтобы добиться большей эффективности ведения подобных кампаний и достичь полной ликвидации филяриоза.

Translated from English version into Russian by Julia Zarubinska-Toepritz, through

TRANSLATORS
WITHOUT BORDERS

Administración masiva de medicamentos para la eliminación de la filariasis linfática en un estado costero de la India: estudio sobre las barreras relacionadas a la cobertura y al cumplimiento

Mohammad A. Hussain, Ashok K. Sitha, Subhashisa Swain, Shridhar Kadam, Sanghamitra Pati

Resumen

Contexto: Se ha fijado como objetivo la eliminación de la filariasis linfática en la India a través de la administración masiva de medicamentos (MDA, por sus siglas en inglés) mediante dietilcarbamacina (DEC) combinado con albendazol (ABZ). Para que la estrategia resulte eficaz, >65% de las personas que viven en zonas endémicas debe estar cubierto y cumplir con la MDA. Tras la campaña MDA 2011 en el distrito endémico de Odisha, realizamos una encuesta para evaluar: (i) el conocimiento sobre filariasis en la comunidad, (ii) la cobertura y el cumplimiento de la MDA desde una perspectiva comunitaria, y (iii) los factores que afectan al cumplimiento, así como las cuestiones operativas relevantes en la realización de las actividades MDA desde la perspectiva del distribuidor de medicamentos.

Métodos: Se seleccionó una muestra de 691 participantes, tanto hombres como mujeres, de más de dos años de edad, a través de un proceso de muestreo estratificado de etapas múltiples y se les entrevistó mediante un cuestionario semiestructurado. Asimismo, se entrevistó igualmente a los distribuidores de medicamentos y al responsable médico a cargo de la MDA para entender algunas de las cuestiones operativas que surgieron durante la MDA.

Resultados: El noventa y nuevo por ciento de los participantes en el estudio recibieron pastillas de DEC y ABZ durante la MDA, de los cuales solo algo más de una cuarta parte llegaron a consumir realmente los medicamentos. La causa de no cumplimiento se debió mayoritariamente al miedo a los efectos secundarios, la falta de conocimientos sobre los beneficios de la MDA y la falta de atención del personal médico en las aldeas. La falta de una formación adecuada de los distribuidores de medicamentos y escasas actividades de comunicación sanitarias antes del inicio de la MDA, así como la ausencia de un seguimiento por parte del personal sanitario tras la MDA fueron algunas de las dificultades operativas encontradas durante la campaña.

Conclusión: Actualmente, la MDA se limita únicamente a la distribución de medicamentos y las cuestiones clave sobre la implementación adecuada, la formación

sanitaria, la gestión de los efectos secundarios y la logística, no están recibiendo suficiente atención. Por ello, resulta fundamental ocuparse de estas cuestiones vinculadas al bajo cumplimiento para conseguir que el programa resulte más eficaz y lograr el objetivo de eliminar la filariasis.

Translated from English version into Spanish by Nazaret Álvarez, through

TRANSLATORS
WITHOUT BORDERS
